# A critical appraisal of integrating family constellation therapy with pharmacological treatment for depression

**DOI:** 10.3389/fpsyt.2026.1904032

**Published:** 2026-09-03

**Authors:** Wenhao Zhong, Mei Rao, Qian Zhou, Zeying Leng, Bing Zhang, Zhijian Lin

**Affiliations:** 1 School of Chinese Materia Medica, Beijing University of Chinese Medicine, Beijing, China; 2 Department of Pharmacy, Longyan First Hospital Affiliated to Fujian Medical University, Longyan, China

**Keywords:** biopsychosocial model, depression, family constellation therapy, family system, integrated treatment, pharmacological treatment, psychosocial intervention, systemic therapy

## Abstract

Depression remains a leading cause of disability worldwide, driven by a complex interplay of biological, psychological, and social factors. While pharmacotherapy, particularly selective serotonin reuptake inhibitors and serotonin-norepinephrine reuptake inhibitors, provides essential symptom control, its limitations—including delayed onset, variable response, and side effects—underscore the need for complementary approaches that address systemic and relational roots of the disorder. This narrative review synthesizes evidence on the integration of antidepressant medication with family constellation therapy (FCT), a systemic, experiential group intervention targeting transgenerational family dynamics. We first outline the core mechanisms and clinical challenges of pharmacotherapy, then examine how adverse family environments, dysfunctional communication patterns, and unresolved familial trauma contribute to depression risk. Next, we present the theoretical foundations and preliminary evidence base for FCT, noting moderate effect sizes for general psychopathology but a critical lack of depression-specific randomized trials. The central argument is that combining pharmacotherapy and FCT aligns with the biopsychosocial model by concurrently stabilizing acute biological symptoms and addressing the family-systemic factors that sustain vulnerability. However, direct comparative evidence is absent, and current support for integration is drawn largely from indirect data on combined psychotherapy and medication. We discuss sequential and parallel implementation models, highlight the urgent need for rigorous randomized controlled trials, and identify practical barriers including limited therapist training and the absence of standardized treatment protocols. In conclusion, the integration of pharmacotherapy with FCT represents a theoretically promising but empirically unvalidated strategy; its clinical utility will depend on the development of testable, manualized protocols and

## 1 Introduction

Depression is a leading global cause of disability and disease burden and presents a formidable challenge in mental healthcare. The primary pharmacological approach to managing this condition involves the use of antidepressant medications (ADMs), which have been steadily prescribed in recent decades (1). These drugs, particularly second-generation agents such as selective serotonin reuptake inhibitors (SSRIs) and serotonin-norepinephrine reuptake inhibitors (SNRIs), function by modulating key neurotransmitter systems, such as serotonin and norepinephrine, and are established as first-line therapies for major depressive disorder (MDD) (1). The selection of a specific agent is a nuanced clinical decision informed by treatment history, patient comorbidities, cost considerations, and risk profile for adverse effects (1). Expert consensus further refines this approach, suggesting that the choice of antidepressant may be tailored to a patient’s predominant symptom profile; for instance, escitalopram or sertraline might be preferred when anxiety is prominent, while duloxetine or venlafaxine could be selected for loss of interest, and mirtazapine for issues such as insomnia or loss of appetite (2). This personalized pharmacological strategy underscores the need for flexibility, considering both the patients’ situational needs and the distinct pharmacotherapeutic profiles of the available medications (2).

Despite their widespread use, pharmacological treatments for depression have significant limitations. A critical issue is the delayed onset of therapeutic effects, which can leave patients vulnerable during the initial weeks of treatment. Furthermore, a substantial proportion of patients do not achieve an adequate response to first-line antidepressants, a condition defined as treatment-resistant depression (TRD), which is associated with a more severe illness course and increased risk of suicide (3). Even when effective, the use of antidepressants is often accompanied by a range of side effects, including sexual dysfunction, which can affect adherence and quality of life (QOL) (4). Perhaps most concerning is the evidence regarding long-term use, as few studies have examined the safety and efficacy of antidepressants beyond two years, and there is an established increased risk of relapse or recurrence upon discontinuation compared to continued use (1). This risk necessitates careful management, with evidence suggesting that gradually tapering the dosage while concurrently providing cognitive behavioral therapy (CBT) can help mitigate withdrawal symptoms and reduce the risk of relapse. The limitations of monoamine-based drugs have spurred intensive research into novel pharmacological targets, including glutamatergic pathways (e.g., N-methyl-D-aspartate [NMDA] and metabotropic glutamate receptors), inflammatory processes, the hypothalamic-pituitary-adrenal axis, and specific G-protein-coupled receptors, in the quest for faster-acting and more effective treatments for MDD.

Concurrently, there is a growing recognition that depression cannot be fully understood or treated through a purely biological lens. The biopsychosocial model provides a more comprehensive framework, emphasizing the intricate interplay between biological vulnerabilities, psychological processes, and social-environmental factors in the etiology and maintenance of the disorder (5). This model is crucial for moving beyond reductionist biomedical discourse and developing holistic interventions (6). Psychological and social determinants are particularly salient, as evidenced by research linking depression to factors such as chronic stress, poor self-esteem, discrimination, financial strain, loneliness, and low social support. For instance, studies on specific populations, such as first-generation Black African immigrants in the United States, have identified discrimination, loneliness, worries about safety, and financial strain as significant biopsychosocial predictors of depression and anxiety symptoms (7). Similarly, in perimenopausal women, a life stage associated with increased vulnerability, the risk factors for depressive symptoms include a history of depression, burdensome menopausal complaints, perceived stress, poor body image, and low self-esteem, along with biological factors such as estradiol fluctuations. This body of evidence strongly suggests that effective treatment must address these multifaceted psychosocial roots, which are often embedded within family systems and interpersonal relationships.

In particular, the family environment plays a pivotal and complex role in depression. Family systems are not merely a backdrop but are active, dynamic entities that can significantly influence an individual’s mental health through patterns of communication, roles, attachments, and transmission of stress or trauma across generations. Dysfunctional family dynamics, conflict, and lack of emotional support are well-established risk factors for the development and perpetuation of depressive disorders. Furthermore, depression can strain family relationships, creating a vicious cycle that impedes the recovery process. Therefore, therapeutic approaches that explicitly target these relational and systemic factors are essential. Although various forms of family therapy exist, one innovative systemic approach is family constellation therapy (FCT). This method operates on the premise that individuals are unconsciously influenced by hidden loyalties and unresolved traumas within their family systems, including those passed down from previous generations. FCT, with its phenomenological focus on transgenerational entanglements and systemic orders, offers a method to directly engage the family risk constellations described in the empirical literature. By making these dynamics visible through experiential group work, the therapy aims to release individuals from entanglement and restore a healthier sense of order and belonging within their familial context. Although rigorous research on its efficacy is still emerging, preliminary studies in naturalistic settings suggest that FCT can be effective in reducing a variety of psychopathological symptoms, including depression and anxiety, and in increasing general well-being, with benefits sustained over a medium-term follow-up period (8). Its brief, group-based format also offers potential advantages in terms of cost-effectiveness and accessibility (8).

**Figure 1:**
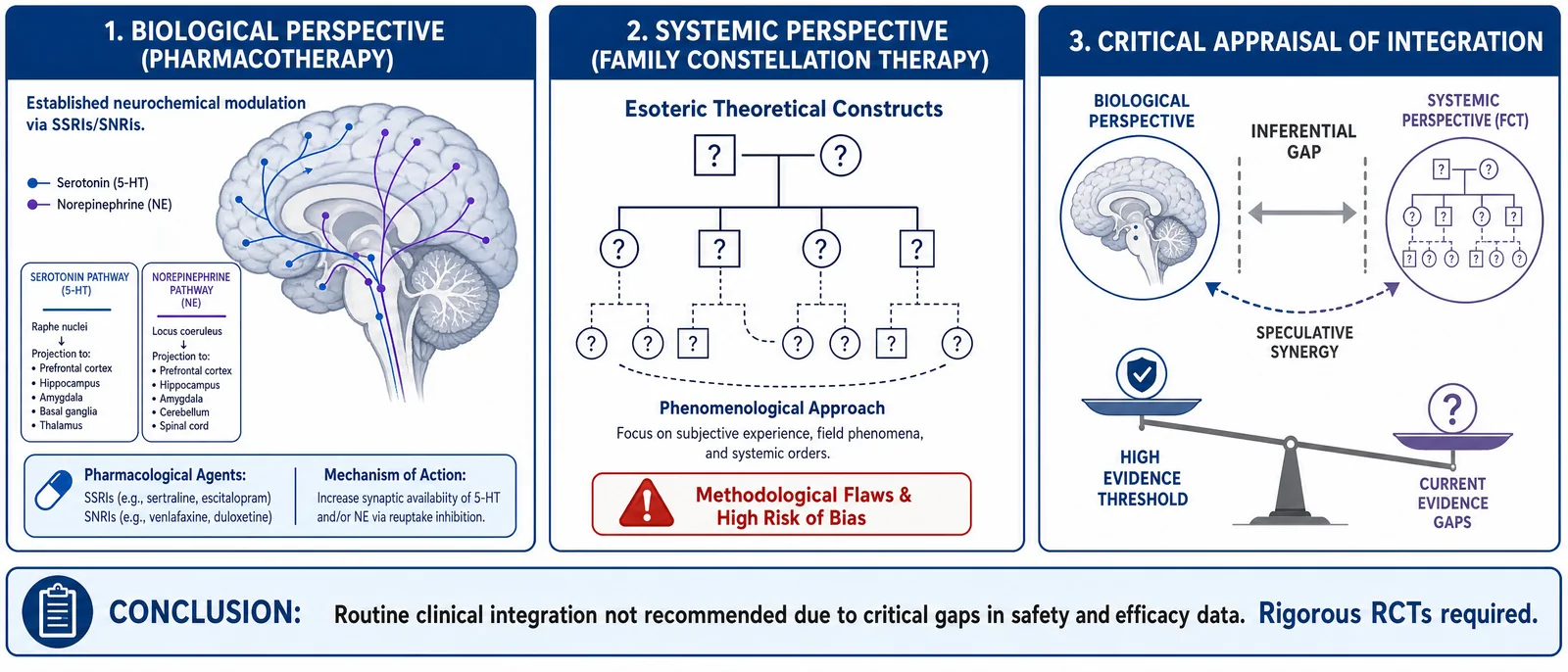
Critical appraisal of integrating pharmacological treatment and family constellation therapy for depression.

Given the complementary strengths and inherent limitations of pharmacological and systemic psychosocial interventions, there is a compelling rationale for exploring integrated treatment strategies. The biopsychosocial model calls for such an integrative approach, as it posits that biological, psychological, and social factors are interconnected and should be addressed concurrently during treatment. In clinical practice, the combination of medication and psychotherapy is the preferred approach for treating severe depression (1). Evidence from network meta-analyses indicates that combined treatment is more effective than either psychotherapy or pharmacotherapy alone in achieving a treatment response and remission (9). This superiority appears to hold across different treatment modalities and is not significantly moderated by baseline depression severity, suggesting its broad applicability (10). Moreover, research suggests that the effects of combined treatment may be enduring, with some studies showing a sustained advantage over pharmacotherapy alone in preventing relapse and recurrence, even after treatment termination (11). The sequential model of treatment, which involves initiating psychotherapy following successful acute-phase pharmacotherapy, has also shown promise in reducing relapse risk by addressing residual symptoms and enhancing psychological well-being (12). This evolving evidence highlights the general value of integrated, patient-centered care for depression. However, the extent to which these benefits apply to FCT specifically—given its esoteric theoretical foundations and the severe methodological limitations of existing studies—remains an open and critical question. In this review, we provide a critical appraisal of the theoretical and empirical basis for integrating FCT with pharmacotherapy, examining whether such integration is currently justifiable based on available evidence. The conceptual framework and key evidentiary limitations considered in this critical appraisal are summarized in **Figure 1**.

## 2 Core mechanisms and clinical practice of pharmacological treatment for depression

### 2.1 Major antidepressant drug targets and classification

The pharmacological landscape of depression treatment is primarily defined by agents that modulate monoaminergic neurotransmission, with SSRIs and SNRIs being the current first-line therapeutic options. These drugs, such as fluoxetine, sertraline, venlafaxine, and duloxetine, exert their antidepressant effects by blocking the presynaptic reuptake transporters for serotonin (5-HT) and, in the case of SNRIs, norepinephrine (NE), thereby increasing the synaptic availability of these monoamines (13). This enhanced monoaminergic signaling is believed to initiate downstream neuroplastic changes, although the precise mechanisms linking acute reuptake inhibition to sustained mood improvement are complex and involve subsequent molecular cascades (14). The clinical utility of SSRIs and SNRIs is tempered by their characteristic profile of adverse effects, including gastrointestinal disturbances, sexual dysfunction, and, for SNRIs, potential effects on heart rate and blood pressure, which can impact patient adherence and QOL (13). Beyond these first-line agents, other pharmacological classes with distinct mechanisms provide alternative and adjunctive strategies. These include the noradrenergic and specific serotonergic antidepressant (NaSSA) mirtazapine, which acts as an antagonist at presynaptic α2-adrenergic receptors and specific serotonin receptors, and the dopamine and norepinephrine reuptake inhibitor (NDRI) bupropion (13). Older classes, such as tricyclic antidepressants (TCAs) and monoamine oxidase inhibitors (MAOIs), while effective, are often reserved for treatment-resistant cases because of their broader side effect profiles and, in the case of MAOIs, significant dietary and drug interaction restrictions (13). The discovery of rapid-acting antidepressants has introduced a paradigm shift, highlighting the glutamatergic system’s critical role. Ketamine and its S-enantiomer, esketamine, primarily function as antagonists of the NMDA receptor (15). This initial blockade triggers a cascade of events, including a surge in glutamate release, subsequent preferential activation of α-amino-3-hydroxy-5-methyl-4-isoxazolepropionic acid (AMPA) receptors, downstream induction of key neurotrophic factors, such as brain-derived neurotrophic factor (BDNF), and activation of the mechanistic target of rapamycin (mTOR) signaling pathway (16). These molecular events promote rapid synaptogenesis and restoration of synaptic connectivity in brain regions such as the prefrontal cortex, which are often compromised by chronic stress, correlating with swift improvements in depressive symptoms observed in both preclinical models and clinical settings (15). Importantly, research suggests that the antidepressant action of ketamine and its enantiomers extends beyond simple NMDA receptor antagonism, involving other systems, such as opioid receptors and potentially anti-inflammatory pathways mediated through transcription factors such as nuclear factor kappa B (NF-κB) (17). Other glutamatergic modulators, including metabotropic glutamate (mGlu) receptor ligands, are also being investigated as potential rapid-acting agents (18). This expansion beyond monoamine-centric models represents a significant breakthrough, particularly for TRD, and underscores the ongoing evolution in our understanding of the neurobiological substrates of antidepressant action (19).

### 2.2 Efficacy evaluation and limitations of pharmacotherapy

The efficacy of antidepressant pharmacotherapy is characterized by significant heterogeneity, with overall response rates typically ranging between 50% and 70%, leaving a substantial proportion of patients with inadequate symptom relief (20). This variability underscores the need for personalized treatment strategies, often involving dose optimization, switching between drug classes, or employing augmentation therapy. A critical limitation of most conventional antidepressants, including SSRIs and SNRIs, is the delayed onset of their therapeutic action, often requiring 2–4 weeks or longer of continuous dosing to achieve a full clinical response (21). This latency period increases the risk of treatment dropout during the initial phases and prolongs patient suffering. In stark contrast, rapid-acting antidepressants, such as ketamine and esketamine, can produce significant antidepressant effects within hours or days, challenging the traditional dogma and offering a crucial alternative for urgent cases, such as those with high suicidal risk (15).

The side effect profiles of antidepressants significantly influence treatment tolerability, adherence, and overall QOL. Common adverse effects associated with SSRIs and SNRIs include gastrointestinal symptoms (nausea and diarrhea), sexual dysfunction (e.g., decreased libido and anorgasmia), weight changes, and either sedative or activating effects (13). Sexual dysfunction is a particularly distressing and frequent side effect that can lead to non-adherence to medication (22). Although newer agents, such as vortioxetine, may have a somewhat improved tolerability profile regarding sexual dysfunction and insomnia, gastrointestinal symptoms remain a concern (13). The risk of severe toxicity from overdose also varies among antidepressants, with agents such as amitriptyline, clomipramine, venlafaxine, and bupropion presenting a higher risk profile than others (23). Furthermore, discontinuation of antidepressants, particularly SSRIs and SNRIs, can lead to a withdrawal syndrome characterized by flu-like symptoms, dizziness, and sensory disturbances, necessitating careful, gradual tapering under medical supervision rather than abrupt cessation (24). Long-term maintenance therapy is crucial for preventing relapse, especially in individuals with recurrent depression, and the decision to continue or discontinue medication must involve a careful risk-benefit assessment shared between the patient and the clinician (20). The exploration of novel mechanisms beyond monoamine reuptake inhibition, such as the modulation of GABAergic systems (e.g., brexanolone and zuranolone), glutamatergic pathways (e.g., mGlu2/3 antagonists), and psychedelic-assisted therapies (e.g., psilocybin), aims to address these limitations by developing agents with superior efficacy, faster onset, and better tolerability (25).

## 3 The pathological role of family system factors in depression

### 3.1 Family environment and depression risk: evidence from epidemiological and longitudinal studies

Epidemiological and longitudinal research provides robust evidence for the significant role of the family environment in shaping the risk of depression across the lifespan. A core finding is the profound impact of parental psychopathology. A network analysis study of adolescents with depression highlights that while childhood trauma experiences, such as emotional abuse and neglect, are the most central environmental factors and primary bridges to depressive symptoms, family and peer risk factors also constitute significant nodes in the risk network (26). This suggests that parental mental health issues create a specific “risk constellation” within the family system. The influence of the family’s emotional climate is particularly potent. Longitudinal data from the Young Finns study demonstrate that a risky emotional family environment in childhood predicts higher levels of pro-inflammatory cytokines (IL-2, IL-6, IFN-γ, TNF-α) in adulthood, which are biomarkers previously linked to depression, indicating a potential biological pathway from early familial stress to later depressive vulnerability (27).

The quality of family functioning and communication patterns is critically linked to the onset and course of depression. Research consistently shows that dysfunctional family environments characterized by high conflict and low cohesion are strong predictors. A study on patients with first-episode psychosis found that a family environment with high emotional over-involvement (EOI) is a significant predictor of psychotic relapse, and a comorbid major depressive episode further increases this risk, underscoring the interplay between family dynamics and depressive symptomatology in severe mental illness (28). Similarly, a longitudinal study of Chinese postgraduate students found that the family environment significantly predicted depressive symptoms over time, with this association being moderated by perceived family support and mediated by psychological resilience (29).

The mechanisms through which the family environment influences depression are multifaceted and often involve socioeconomic and relational factors. Low family socioeconomic status (SES) can elevate the risk of depression by limiting resources and increasing chronic stressors. A study in rural China found that family economic conditions influence children’s and adolescents’ mental health outcomes, loneliness, depression, and Internet addiction, primarily through pathways involving the primary caregiver’s emotional well-being, hostile parenting styles, and poor family communication (30). This highlights how economic disadvantage can translate into adverse parenting practices and strained family interactions, which in turn become direct risk factors for depression. Specific dimensions of the family environment, such as cohesion, conflict, and expressiveness, have been directly correlated with depressive symptoms in diverse populations, including military recruits, where aspects such as family entertainment and organization showed negative correlations with depression scores (31). The protective role of a positive family climate is also evident. For instance, higher baseline family cohesion and lower family conflict are independent predictors of better treatment response to combined repetitive transcranial magnetic stimulation (rTMS) and group therapy in adolescents with MDD (32).

Furthermore, patterns that combine both family and school environments are highly informative. Latent profile analysis in a large adolescent sample identified that profiles characterized by family dysfunction and poor school climate were associated with higher subsequent depressive symptoms, mediated by lower resilience, compared to the “good family function-good school climate” group (33). This cumulative risk model is supported by research showing that for adolescents from Mexican immigrant families, specific familial factors, such as high maternal hostility, low cultural socialization, and low parentification experiences during adolescence, exacerbate the association between depressive symptoms and shorter telomere length in late adolescence, a marker of cellular aging (34). Even in older adults, the built environment can interact with family support; living in areas with a higher percentage of green spaces was found to mitigate the negative effects of lower levels of family support on depression among community-dwelling older adults in urban China (35). Network analysis in adolescents with MDD confirmed the intricate interplay, identifying nodes such as emotional abuse, emotional neglect, family cohesion, and family control as among the most central symptoms in a network connecting childhood trauma, family environment, and depressive symptoms (36). Collectively, these findings indicate that a cluster of adverse family factors—including high EOI, conflict, low cohesion, and childhood maltreatment—forms a potent familial risk profile that may serve as a direct target for systemic interventions.

### 3.2 Intergenerational transmission and unresolved family trauma

The concept of intergenerational transmission posits that unresolved familial issues, including trauma, secrets, and dysfunctional relational patterns, can unconsciously influence the psychological well-being of subsequent generations, often manifesting as symptoms such as depression, unexplained guilt, or identity confusion. While current empirical studies do not explicitly test the theoretical constructs of FCT regarding “secrets” or “excluded members,” they offer substantial empirical support for the broader mechanisms of familial transmission of risk, particularly through genetic predispositions, shared family environments, and the psychological impact of parental mental illness and childhood adversity. A foundational element is the well-replicated risk associated with a history of depression in the family. Studies have consistently shown that parental depression is a potent risk factor for offspring. Longitudinal research on young people with a parent with recurrent MDD identified two trajectory classes: a childhood-emerging class (25%) with persistent depressive disorder from age 12.5 years and an adulthood-emerging class (75%) (37). This suggests variability in the developmental course but underscores the high risk of developing MDD. Transmission involves both genetic and environmental factors. Polygenic risk scores (PRS) for MDD (MDD-PGS) and family history of depression are associated with depressive trajectories in adolescents. A study using the ABCD cohort found that MDD-PGS and family conflict predicted higher intercept levels of depressive symptoms and increased the odds of higher-risk trajectories among White, Latinx, and Black youth, with evidence of gene-environment correlation (rGE) through family conflict, specifically in White youth (38). This indicates that genetic predispositions may be expressed within or even shape conflictual family environments. Furthermore, impaired inhibition prospectively predicted greater anxiety and depressive symptoms eight years later, specifically in adolescents at high familial risk for MDD, highlighting a potential cognitive endophenotype that interacts with familial vulnerability (39).

The transmission of risk is also mediated by the quality of the family’s emotional environment and exposure to childhood trauma, which can be seen as forms of unresolved familial stress. A risky emotional family environment in childhood not only predicts depression-related inflammatory markers in adulthood (27) but also interacts with other risk factors. For instance, the association between depressive symptoms and shorter telomere length in Mexican-origin youth was strongest for those who experienced high maternal hostility or low cultural socialization during adolescence (34). This highlights how negative parenting behaviors, potentially stemming from a parent’s unresolved issues, can biologically embed stress in the next generation. Childhood trauma, which often occurs within the family context, is a critical mediator. Network analysis in adolescents with MDD placed emotional abuse and emotional neglect as central nodes and among the strongest bridges linking the broader environment to depressive symptoms (26, 36). Such maltreatment experiences represent profound failures in the caregiving environment that can lead to persistent emotional dysregulation and depression. Relational patterns within the family, such as systemic enmeshment or alienation, are crucial. Studies have shown that family dysfunction, encompassing poor communication, high conflict, and low cohesion, is a robust correlate and predictor of depression in all ages. For example, in postpartum women, experiencing two or more family conflicts per week was a significant risk factor for postpartum depressive symptoms, whereas good family function was protective (40). Similarly, in adolescents, the family emotional environment (e.g., low emotional support) was linked to depressive symptoms through mediating pathways involving Internet addiction and peer relationships, whereas the family economic environment was not directly associated (41). This underscores the primacy of emotional factors over economic factors in the familial transmission of depression risk. The burden of caregiving within families can also create a cycle of distress. Caregivers of hemodialysis patients face high levels of burden and poor sleep quality, with a significant proportion showing depressive symptoms (42), which could subsequently affect their parenting or relational capacities. In late-life depression, neuroticism mediates the relationship between sex, family history, history of depressive disorders, and comorbid anxiety disorders, suggesting that personality traits, which may have familial and genetic underpinnings, contribute to the clinical presentation (43). Finally, the role of the family environment as a moderator is evident; harmonious family relationships were found to significantly mitigate the risk of depression in children with disorders of gut-brain interaction, acting as a protective factor against the psychological impact of chronic illness (44). These empirical findings collectively illustrate the complex intergenerational transmission of depression risk through intertwined genetic vulnerabilities, exposure to adverse childhood experiences within the family, and the enduring impact of dysfunctional family dynamics, providing a scientific framework that aligns with clinical observations of unresolved family trauma affecting subsequent generations. Beyond genetic predispositions and family environment, epigenetic mechanisms have emerged as a critical biological bridge for intergenerational trauma transmission. Epigenetic modifications, particularly DNA methylation, regulate gene expression without altering the DNA sequence and can be inherited. For instance, childhood adversity is associated with increased methylation of the glucocorticoid receptor gene (NR3C1), a key regulator of the hypothalamic-pituitary-adrenal (HPA) axis, thereby elevating depression risk (76). A recent systematic review confirmed sex-specific NR3C1 methylation patterns and neuroendocrine dysregulation extending across generations, often manifesting as treatment-resistant depression (77). Similarly, extreme parental trauma has been shown to induce intergenerational changes in FKBP5 methylation, another crucial stress-regulation gene, observable in both exposed parents and their offspring (78). The transmission of perinatal depression also involves epigenetic changes in glucocorticoid, oxytocin, and immune systems (79). However, while animal models provide robust mechanistic evidence, the extent to which these epigenetic mechanisms underlie intergenerational effects in humans remains a hypothesis requiring further prospective, multi-generational studies (80). Taken together, this epigenetic framework provides a plausible, though incompletely characterized, biological rationale for family-based interventions targeting transgenerational trauma.

Despite the compelling epidemiological and biological evidence linking family adversity to depression, a critical inferential gap remains regarding the specific theoretical constructs of FCT. A recent meta-analysis of 30 randomized controlled trials confirmed that systemic therapy broadly yields larger improvements in depressive symptoms compared to no-treatment controls (Hedges’ g = 1.09) (81). It should be noted that this large effect size was derived primarily from comparisons against no-treatment or waitlist controls, which typically inflates effect estimates compared to active controls. However, it is crucial to recognize that epidemiological findings regarding family risk factors cannot be legitimately extrapolated to validate FCT’s specific metatheories. Evidence-based systemic models are grounded in observable relational dynamics, whereas FCT relies on esoteric phenomenological constructs—such as ‘orders of love’, ‘representative perception’, and transgenerational ‘entanglements’—that are not empirically validated by current epidemiological or epigenetic data. Furthermore, critical analyses explicitly characterize FCT’s metatheories as belonging to the realm of Western esotericism, emphasizing its spiritual and mystical nature rather than any scientifically testable foundation (45). Similarly, recent theoretical appraisals note that FCT operates more as a quasi-religious ritual than a conventional psychological intervention (46). Therefore, while the existing literature provides a robust rationale for systemic family interventions in general, it strictly does not empirically validate the unique, esoteric mechanisms proposed by FCT.

## 4 Theoretical foundations of family constellation therapy and its application in depression

### 4.1 Core principles and operational procedures of FCT

FCT, developed by Bert Hellinger, is a brief group-based psychotherapeutic intervention rooted in systemic family therapy theory. Its foundational principle posits that an individual is an integral part of a larger family system, and psychological symptoms, such as depression, may manifest as expressions of underlying systemic imbalances or disturbances within this network (45). The therapeutic process operationalizes this systemic view through a distinctive method known as “representative perception.” In a group setting, the client (or focus individual) selects other group members to represent key figures from their family system, such as parents, siblings, or even abstract concepts like “the illness” or “fate.” These representatives, without prior knowledge of the individuals they represent, are positioned spatially within the room according to the client’s internal image. From a systemic-phenomenological perspective, representatives frequently describe embodied sensations, emotions, or impulses that appear to reflect the client’s internalized family representations, thereby offering a non-cognitive map of relational tensions and emotional forces at play within the client’s familial field (46). However, the precise psychological mechanisms underlying these experiences remain under investigation. This process aims to make invisible systemic tensions visible.

Within this esoteric framework, the core therapeutic work involves revealing hidden dynamics and restoring what Hellinger termed the “orders of love”—fundamental systemic principles concerning belonging, hierarchy (e.g., respecting the generational order), and balance in giving and taking. The constellation often uncovers suppressed conflicts, misplaced loyalties (where a child unconsciously carries a parent’s fate or guilt), exclusions (of forgotten or ostracized family members), or violations of these orders, which are theorized to be the roots of an individual’s emotional distress (45). For instance, a client’s depression might be linked to an unconscious identification with a deceased, marginalized, or suffering ancestor. The therapist guides the process by observing the representatives’ reactions and spatial configuration, interpreting these as indicators of systemic entanglements in the family. From a phenomenological perspective, the goal is to facilitate a resolution process by carefully adjusting the positions of the representatives, verbally acknowledging excluded members, facilitating symbolic dialogue between representatives, or performing ritualistic acts of respect. These interventions are designed to help the system move towards a state of greater balance and harmony, allowing the individual to be released from carrying burdens that are not rightfully theirs, thereby alleviating symptoms such as depression by restoring a sense of rightful place and peace within the familial soul (46). The entire procedure is phenomenological and experiential, prioritizing felt sense and emergent insights over cognitive analysis.

### 4.2 Intervention focus and empirical exploration for depression

When applied to depression, FCT targets specific systemic roots believed to underlie this disorder. The primary focus of the intervention is on processing unresolved loss and facilitating mourning. The therapy posits that depressive symptoms can stem from incomplete grieving processes within the family history, in which a client may be unconsciously “entangled” with or loyal to a deceased family member. By bringing representatives of the deceased and the living into the constellation, the therapy aims to help the client visually and emotionally confront frozen grief. Through guided acknowledgment, expression of respect, and symbolic completion of farewells, the process seeks to release the depressive energy tied to this identification, allowing the client to live their life fully (46). This work on transgenerational trauma and unattended sorrow is central to addressing what is perceived as energetic or emotional stagnation that manifests as depression.

Another critical focus is on reconstructing a sense of belonging and correcting the systemic order. In FCT, depression is often viewed as a consequence of a disturbed sense of belonging within the family system, potentially arising from events such as the premature death of a parent, abortion, adoption, or family secrets. The therapy assists the client in finding their “rightful place” within the family hierarchy and network. By visually restructuring the family system in the constellation, ensuring that parents are given their due place of respect, children are allowed to be children, and excluded members are reintegrated through acknowledgment, the intervention aims to restore a fundamental sense of security and belonging (45). This re-establishment of order is theorized to alleviate feelings of existential loneliness, worthlessness, and the burden of misplaced responsibility that frequently accompany depressive states, fostering a feeling of being supported by the larger family system.

Regarding preliminary efficacy and mechanism exploration, empirical evidence for FCT remains severely limited in both quantity and methodological quality compared to established psychotherapies. A systematic review synthesizing data up to 2020 highlighted significant methodological flaws across the literature, including a pervasive lack of active control groups, reliance on subjective self-reporting, and highly heterogeneous samples (47). While this meta-analysis reported a moderate effect size (Hedges’ g = 0.531) for reducing general psychopathological symptoms, this figure must be interpreted with extreme caution given the high risk of bias in the primary studies (47). More recent evidence further underscores these limitations; a pre-registered RCT investigating a pandemic-adjusted version of FCT in the general population found that while the intervention group showed initial improvements in overall psychopathology, the magnitude of these improvements was small and largely disappeared by the 6-month follow-up (48). Although some naturalistic studies report sustained benefits in psychopathological symptoms and quality of life (8), the lack of rigorous controlled designs prevents any definitive conclusions regarding FCT’s specific efficacy. Crucially, a comprehensive 2026 systematic review and meta-analysis evaluating FCT across all clinical conditions concluded that there is currently no reliable evidence to support its use for any condition (84). The authors emphasized that all included trials were at a high risk of bias, resulting in very low-certainty evidence, and raised significant concerns regarding potential adverse effects (84). It should be noted that this systematic review is currently a preprint and has not yet undergone peer review; therefore, while it provides a rigorous synthesis of the existing literature, its findings should be interpreted as preliminary and not used to definitively guide clinical practice. Nevertheless, it strongly reinforces the critical need for methodological rigor before any clinical implementation of FCT. This highlights that procedural fidelity and context may significantly impact outcomes, and underscores the need for a more critical evaluation of FCT’s clinical readiness. The proposed mechanisms of action are multifaceted and align with its focus: FCT may promote emotional catharsis and release during the intense experiential process, facilitate cognitive restructuring by providing a new systemic narrative for personal suffering, enhance self-differentiation by clarifying boundaries within the family system, and strengthen perceived systemic support (8). Its effects may be mediated through changes in the internal working models of family relationships and improved emotional processing. Tolerability data suggest that iatrogenic effects (e.g., temporary distress) are comparable to other psychotherapies, occurring in a small minority (≈5–8%) of participants (8, 47). Overall, while preliminary evidence points to FCT’s potential effectiveness and safety for depressive symptoms, the field urgently requires more high-quality, rigorous RCTs to firmly establish its efficacy and elucidate its specific therapeutic mechanisms.

## 5 Integration strategies of pharmacological treatment and FCT

### 5.1 Rationale and synergistic effects of integrated treatment

It is important to note that no RCT has yet directly compared combined pharmacotherapy and FCT against either modality alone; therefore, the following discussion of synergistic mechanisms is primarily informed by general principles of combined treatment for depression and the preliminary evidence for FCT. Nevertheless, the integration of pharmacological and psychotherapeutic interventions for MDD is strongly supported by a complementary rationale that addresses both acute symptom control and underlying psychosocial etiologies, thereby offering a synergistic, multi-target approach aligned with the biopsychosocial model of MDD. First, this combination leverages the distinct temporal and mechanistic advantages of each modality. Pharmacological treatments, primarily ADMs, act on neurobiological pathways to rapidly stabilize severe biological symptoms, such as vegetative symptoms, during a major depressive episode (49). This biological stabilization is crucial because it creates the necessary emotional stability and psychological space for patients to effectively engage in deeper, exploratory psychotherapies (50). In contrast, psychotherapies, including various evidence-based forms such as CBT, interpersonal psychotherapy (IPT), and psychodynamic therapy, target the psychosocial, cognitive, and systemic relational factors that often underlie and perpetuate depressive disorders (49, 51). This division of labor is fundamentally complementary. While medication manages the acute biological burden, psychotherapy works to process potential psychological causes, which may contribute to a reduced risk of relapse and promote more durable recovery (52). A network meta-analysis confirmed that for moderate-to-severe depression, most psychotherapies combined with medication are superior to psychotherapy alone, highlighting the additive benefits in more severe cases (53). A comprehensive meta-analysis demonstrated that combined treatment has a moderate effect on depression compared with pill placebo, and is significantly more effective than pharmacotherapy or psychotherapy alone (82). Similarly, another review concluded that combined treatment offers optimal effectiveness, especially for moderate and severe symptoms (83). However, it is crucial to emphasize that this strong evidence for synergistic effects is derived exclusively from established, evidence-based psychotherapies, such as cognitive behavioral therapy (CBT) and interpersonal psychotherapy (IPT) (53, 82). Current network meta-analyses of combination therapies explicitly exclude unproven modalities (53), and major clinical guidelines reserve combination recommendations solely for empirically supported psychological treatments (49). FCT has not been included in these analyses nor endorsed by clinical guidelines. Therefore, extrapolating the synergistic benefits observed with CBT or IPT to FCT when combined with pharmacotherapy is entirely theoretical and highly speculative. The rationale for integrating pharmacotherapy with FCT remains a theoretical proposition based the biopsychosocial model, rather than an empirically proven synergistic integration. This synergy is particularly relevant for TRD, where psychotherapy addresses the hazards of polypharmacy and explores resistance and neural mechanisms beyond purely pharmacological actions (51).

Second, integrated treatment embodies a true multi-target intervention strategy. Pharmacotherapy primarily modulates neurotransmitter systems (e.g., serotonin and norepinephrine) and neurosteroid actions on receptors such as GABA-A and NMDA to alleviate core depressive symptoms (49, 54). Conversely, psychotherapeutic approaches intervene at the psychological, behavioral, and social system levels of the individual. For instance, therapies may aim to change maladaptive cognitive patterns, improve interpersonal functioning, resolve intrapsychic conflicts, or address family system dynamics, as suggested by the context of FCT (51, 53). By combining these modalities, an integrated scheme achieves comprehensive coverage across all dimensions of the biopsychosocial model of depression, potentially leading to more robust and holistic outcomes than monotherapy (49). Evidence from a quasi-experimental study on chronic depression suggests that while a psychotherapeutic inpatient program was effective regardless of ADM continuation, the combination is often preferred, especially for more severe or chronic presentations (55). Furthermore, machine learning studies aiming to predict individual outcomes of psychotherapy versus medication underscore the existence of variable individual responses, implying that an integrated approach could be personalized to cover multiple vulnerability pathways simultaneously (56).

Finally, integration can significantly enhance treatment adherence and overall efficacy by improving patient engagement and reducing the stigma associated with mental illness. When patients understand that their symptoms may be connected to their psychological background or family systems, internalized stigma may be reduced, and a greater sense of agency and meaning in the recovery process may be fostered (57). This understanding increases identification with and commitment to the overall treatment plan. Research indicates that aligning treatment with patient preferences is critical for adherence; mismatches between patient preferences for psychotherapy and the treatment received are associated with worse adherence (58). An integrated approach that offers both biological and psychological explanations and interventions may better accommodate diverse patient beliefs and preferences, thereby boosting engagement. For example, patients who strongly prefer psychotherapy are more likely to adhere to it if they receive it (58). Moreover, in complex comorbid conditions, such as depression in patients with coronary artery disease, chronic kidney disease, or post-stroke, integrated care combining pharmacological and psychological interventions is frequently studied and recommended, acknowledging the need to address both the medical and psychological facets of the illness to improve outcomes, such as mortality, cardiac events, and functional recovery (59–61). Thus, the rationale for integration is rooted in creating a synergistic, multidimensional, and patient-centered framework that maximizes the therapeutic potential across the spectrum of depressive disorders.

### 5.2 Clinical implementation models and challenges

The clinical implementation of integrated pharmacological and psychotherapeutic interventions for depression can be operationalized through several models, primarily sequential and parallel integration, each with specific indications and logistical requirements. However, all face significant challenges, including a lack of standardized protocols, insufficient evidence for specific therapies, and the need for cross-disciplinary training in the field.

Crucially, rather than advocating for the universal application of family systemic interventions like FCT across all depressed populations, clinical assessment must prioritize patient stratification. Recent large-scale cohort studies demonstrate a strong familial susceptibility to treatment-resistant depression (TRD), where first-degree relatives of TRD patients have a more than nine-fold increased risk of developing TRD themselves (85). Consequently, patients with a strong family history of depression or TRD may require more intensive, multi-modal interventions earlier in their treatment course (85). Furthermore, clinical frameworks for TRD increasingly recognize that treatment resistance is not solely a pharmacological failure but is deeply intertwined with psychosocial factors, including family conflict, early life adversity, and interpersonal difficulties (86). Therefore, the theoretical target population for exploring systemic family interventions should be strictly defined: it is not intended for first-episode, uncomplicated depression, but rather as a potential adjunctive exploration for patients with TRD or difficult-to-treat depression (DTD) who present with prominent, unresolved familial trauma or severe systemic relational dysfunction (86, 87). Critically, however, a fundamental safety paradox must be acknowledged: while patients with TRD and profound familial trauma may theoretically be the most in need of deep systemic intervention, they are also the most psychologically vulnerable. For exploratory, emotionally intense, and currently unvalidated interventions such as FCT, greater clinical severity raises—not lowers—the required safety and efficacy threshold. In the absence of rigorous safety data, directing the most vulnerable patients toward such approaches carries a substantial risk of iatrogenic distress and cannot be endorsed.

Within this stratified context, the sequential integration model involves a staged approach in which pharmacological intervention is prioritized during the acute phase to control core and severe depressive symptoms (49). Once the patient’s condition is stabilized biologically and emotionally, a psychotherapeutic intervention, such as FCT aimed at deep exploration and personality growth, is introduced. This model is supported by evidence suggesting that medication can provide the necessary symptomatic relief for patients to engage meaningfully in psychotherapy (50). For instance, in chronic depression, an intensive psychotherapeutic program can be effective, and the decision to continue or discontinue ADM during such therapy can be tailored based on individual assessment, suggesting a flexible, sequential, or overlapping approach (55). This model is pragmatic in standard clinical settings, where immediate symptom reduction is the priority, and aligns with findings that the combination is more effective than either treatment alone for moderate-to-severe depression (53).

In contrast, the parallel integration model involves simultaneous pharmacotherapy and psychotherapy from the outset of treatment. This can be achieved through collaboration between professionals from different backgrounds (e.g., a psychiatrist managing medication and a psychologist providing psychotherapy) or by a single clinician trained in both modalities (62). This model is particularly relevant for conditions in which psychological and biological factors are intensely intertwined from the start, such as depression comorbid with chronic medical conditions including stroke, coronary artery disease, or traumatic brain injury (59, 61, 63). Research on combined treatments for post-stroke depression often evaluates pharmacological and psychological interventions delivered concurrently (61). A review of non-invasive brain stimulation combined with medication or psychotherapy for MDD exemplifies parallel integration, in which treatments are administered together to potentially enhance efficacy (62). This model requires careful coordination to ensure that the treatments are synergistic and not contradictory.

Despite these potential benefits, the implementation of integrated care faces substantial challenges. A primary challenge is the lack of standardized, empirically validated protocols for integrating specific psychotherapies, such as FCT, with pharmacotherapy (53). While evidence supports the general principle of combining psychotherapy and medication, the efficacy evidence for FCT within a robust, contemporary evidence-based framework remains limited and requires strengthening (53, 64). This is part of a broader issue in psychotherapy research, in which trial comparability and control conditions can be problematic (64). Second, effective integration requires therapists to possess cross-disciplinary knowledge and skills. They must understand the neurobiological aspects of depression and pharmacodynamics to collaborate with prescribers or manage medications themselves, while also being proficient in complex psychotherapeutic techniques (51). This training gap is a significant barrier. Third, a major clinical challenge is the precise matching of patient characteristics to the optimal treatment combinations and sequences. Factors such as depression severity, chronicity, comorbid personality pathology, and patient preferences significantly influence outcomes (52, 56, 58). For example, patients with severe depression or personality pathology may have different long-term outcomes, with some evidence suggesting that psychotherapy may be superior to medication in the long term (52). Machine learning approaches are being explored to predict individual patient responses to combined treatments, which could guide personalized matching in the future (65). Furthermore, practical barriers exist, such as the low utilization rates of evidence-based psychotherapies compared to medication in large healthcare systems, often due to high patient volume, clinician capacity, and patient factors such as comorbidities (66). Finally, in specific populations such as youth, the comparability of medication and psychotherapy trials is questioned because of differences in participant characteristics and control conditions, complicating the development of clear, integrative guidelines (64). Addressing these challenges requires concerted research efforts to develop and test specific integrated protocols, investment in cross-disciplinary training programs, and the development of decision-support tools to facilitate personalized treatment planning.

## 6 Discussion

### 6.1 Future research directions and clinical implications

Rigorous clinical trials: There is a critical need for well-designed RCTs to compare the efficacy of integrated interventions against single-modality treatments, either pharmacological or psychological alone, for acute-phase depression, long-term relapse prevention, and social functional recovery. Foundational evidence for integrated approaches often stems from studies of specific combinations, such as digital tools used in psychotherapy or collaborative care models. For instance, an updated meta-analysis of 12 RCTs confirmed the effectiveness of the tailored, integrative Internet intervention “deprexis” for reducing depressive symptoms, demonstrating a medium effect size that was consistent across various study qualities and participant severities (67). Similarly, integrated behavioral interventions for comorbid conditions, such as obesity and depression, have shown varied but promising results in RCTs, with some studies reporting greater improvements in both conditions than in usual care (68). However, this field faces significant methodological challenges. As highlighted in psychotherapy research, RCTs for complex multicomponent interventions, such as integrated treatments, cannot utilize double-blinding and test composite packages, which may limit the direct comparability of findings to pharmacological trials (69). This underscores the necessity for trials that meticulously define the components of ‘integrated care,’ whether it combines pharmacotherapy with a specific psychotherapy model, FCT, or digital support, and employs active comparators. For example, a study on cognitive therapy enhanced with a memory support intervention showed a significant reduction in depression severity at a 6-month follow-up compared to cognitive therapy as usual, highlighting the potential value of integrating specific supportive techniques (70). Future trials must be designed with sufficient power, adequate follow-up periods (beyond 12 months to assess relapse), and standardized outcome measures that capture not only symptom reduction but also functional and QOL outcomes to robustly establish the comparative effectiveness of integrated strategies in treating depression.

Exploring Biomarkers and Predictive Models: Advancing personalized medicine for depression requires the identification of biomarkers that can predict differential responses to pharmacological, psychosocial, or integrated interventions. This involves leveraging tools from neuroimaging, genetics, and immunology. Although current clinical guidelines do not yet incorporate biomarker-driven treatment selection for systemic therapies, the highly heterogeneous nature of treatment response underscores the critical rationale for such research. For example, studies on integrated interventions for comorbid depression and obesity have shown varied patient trajectories, with some participants achieving substantial improvements in both domains while others show minimal change, suggesting underlying biological or psychological moderators (71). Research in other areas, such as the response to an integrated collaborative care intervention for obesity and depression, has found that differential outcomes are associated with distinct clusters of self-reported COVID-19 impact (e.g., mental health vs. economic impact), hinting at how external stressors and possibly their associated biological correlates (e.g., inflammation) might influence treatment success (72). Future research should systematically collect biospecimens and neuroimaging data within RCTs of integrated therapies to correlate treatment outcomes with baseline measures of inflammation (e.g., cytokines), neural circuit activity (e.g., in the default mode or emotion regulation network), and genetic polymorphisms. The goal is to move beyond a one-size-fits-all approach, enabling clinicians to use objective markers to guide whether a patient might benefit more from an SSRI, a family systems intervention, or a combination thereof, thereby optimizing resource allocation and improving individual patient outcomes.

Mechanistic Research: Understanding the specific psychophysiological mechanisms through which psychosocial components, such as FCT, exert their therapeutic effects is essential for refining integrated interventions. This requires employing methods from psychology and neuroscience to trace the pathway from therapeutic interventions to neural and systemic changes. Although the existing literature focuses on integrated interventions broadly rather than specifically on FCT’s mechanisms, it highlights the importance of understanding change processes. For instance, the proposed integrative framework linking family dynamics to childhood depression posits that dimensions such as communication and cohesion shape parent–child attachment security, which serves as the central mediating pathway to depressive symptoms (73). This suggests a testable mechanistic model in which family therapy improves depression by repairing attachment bonds, a process that can be measured through changes in relational behaviors, stress physiology (e.g., cortisol levels), and associated brain networks. Furthermore, studies on other integrative modalities offer useful analogies. Research on combined yoga and dietary interventions for MDD found significant improvements in autonomic regulation (heart rate variability) and cognitive function alongside reduced depression scores, pointing to potential mechanisms involving the parasympathetic nervous system and neurocognitive pathways (74). Similarly, the integration of passive sensing technology (e.g., GPS, activity tracking) into psychological interventions for maternal depression allowed for the observation of behavioral correlates of improvement, such as increased movement away from home, providing an objective, mechanistic lens on behavioral activation (75). Applied to family therapy, mechanistic studies could use functional MRI to investigate changes in the brain’s default mode network (linked to self-referential thought) or salience network following interventions that alter family narratives and roles. Psychophysiological measures can assess how therapy modulates the hypothalamic–pituitary–adrenal axis response to family-related stressors. By elucidating these mechanisms, research can identify the active ingredients of complex interventions, allowing for their optimization and more targeted application in integrated treatment plans.

## 7 Conclusions

The evolution of depression treatment from a purely biomedical model to an integrated biopsychosocial framework represents a significant paradigm shift in modern psychiatry. This transition acknowledges the multifaceted nature of depression, recognizing that its etiology and maintenance are seldom attributable to a single factor. While pharmacological interventions remain a cornerstone for rapid symptom alleviation, their limitations have catalyzed the search for complementary approaches. The integration of Family Constellation Therapy (FCT) into this landscape offers a systemically oriented perspective that posits symptoms as expressions of unresolved familial entanglements. However, we must explicitly acknowledge the significant inferential gap between general epidemiological evidence of familial risk and the esoteric theoretical constructs unique to FCT. Furthermore, while combined pharmacotherapy and established psychotherapies (like CBT or IPT) show proven synergistic effects, assuming identical benefits for FCT remains highly speculative. FCT’s clinical readiness is heavily constrained by severe methodological limitations in existing studies, high risk of bias, and documented concerns regarding iatrogenic distress in vulnerable populations (47, 84). Given these critical gaps in safety and efficacy data, routine clinical referral for FCT cannot be recommended at this time. Ultimately, the integration of pharmacotherapy with systemic family interventions represents a theoretically intriguing but empirically unvalidated strategy. Future research should abandon a ‘one-size-fits-all’ approach and instead focus on highly stratified subpopulations, specifically patients with treatment-resistant depression characterized by profound familial trauma (85, 86). Its future clinical utility strictly hinges on the development of manualized protocols and robust, rigorous randomized controlled trials to substantiate its safety, unique mechanisms, and efficacy, prior to any clinical implementation.
